# Knowledge Production and Learning for Sustainable Landscapes: Forewords by the Researchers and Stakeholders

**DOI:** 10.1007/s13280-012-0371-5

**Published:** 2013-03-10

**Authors:** Per Angelstam, Marine Elbakidze, Robert Axelsson, Niels Elers Koch, Tatiana I. Tyupenko, Alexandr N. Mariev, Lennart Myhrman

**Affiliations:** 1 Faculty of Forest Sciences, School for Forest Management, Swedish University of Agricultural Sciences, P.O. Box 43, 730 91 Skinnskatteberg, Sweden; 2 Faculty of Forest Sciences, School for Forest Management, Swedish University of Agricultural Sciences, P.O. Box 43, 739 21 Skinnskatteberg, Sweden; 3 Forest & Landscape Denmark, University of Copenhagen, Rolighedsvej 23, 1958 Frederiksberg C, Denmark; 4 Russian Federal Forestry Service, Pyatnitskaya Street 59/19, 115184 Moscow, Russian Federation; 5 Gamla Nåsvägen 10A, 770 10 Fredriksberg, Sweden; 6 LEADER Bergslagen, Box 101, 739 22 Skinnskatteberg, Sweden; 7 Ministry of Natural Resources and Environmental Protection of the Komi Republic, 108a Internatsionalnaya Street, Syktyvkar, Komi Republic Russian Federation

**Keywords:** Sustainability science, Transdisciplinary, Ecosystem services, Natural experiment, Europe, Russia

## Abstract

This special issue of AMBIO presents a new approach to sustainability science that goes beyond interdisciplinary research. Using coupled natural and human systems, or landscapes, as multiple case studies in Europe’s East and West knowledge production and learning toward transdisciplinary research was applied in Sweden, countries in Central and Eastern Europe, and Russia. First, the research group Forest-Landscape-Society summarizes the research program (2005–2012) behind this special issue of AMBIO and its development to participate in transdisciplinary research. Second, stakeholders at multiple levels provide their views on the new approach presented and reported.

## Introduction

Landscapes deliver goods, functions, and values on which human welfare and quality of life are built. The term ecosystem services capture this, and it was developed to communicate nature as a fixed stock of natural capital that can sustain a limited flow of services (Norgaard 2010). However, given the diverse and often conflicting policy visions in multiple natural resource sectors and the fact that sustainable development is a multi-stakeholder social learning process at local, regional, national, and international levels of governance with sustainability as the ultimate goal, there are many challenges to deal with. The current increased economic interest in natural resources implies intensified use. This applies both to the European continent’s East and West (Angelstam et al. 2011a). However, there are also large differences among landscapes in different countries and regions, which can be used as a natural experiment in social–ecological systems. In the East, countries with transition economies (Myant and Drahokoupil 2010) share several challenges regarding the reformation of their natural resource use, governance, and management as a part of the transition process from planned to market economy (Holopainen et al. 2006; Nysten-Haarala 2009). The West countries are challenged with landscape restoration for biodiversity conservation (e.g., Angelstam et al. 2011b) and human well-being (MEA 2005), especially in the light of the increasing demands of natural resources (Andersson et al. 2012). In addition, in both East and West, there is also a desire to secure livelihoods and human well-being in rural areas (O’Brien and Patsiorkovsky 2006).

There are two objectives of this suite of forewords. First, the research group Forest-Landscape-Society summarizes the research program (2005–2012) behind this special issue of AMBIO, and the group’s development process to participate in transdisciplinary research aiming at knowledge production and learning to support the development toward sustainable landscapes (see Haines-Young 2000; Antrop 2006). Second, a group of stakeholders at multiple levels provide their views on the philosophy of knowledge production and learning toward transdisciplinary research presented in this issue.

## Summarizing a Research Group’s Journey 2005–2012

Using a research methodology with case studies in multiple landscapes along distinct gradients in ecological and social systems across the European continent, from the Russian Federation in the East, via Eastern and Central Europe, to the West (e.g., Best 2009; Angelstam et al. 2011a), our aim is to produce novel knowledge and encourage learning for sustainable development and sustainability in social–ecological systems (Angelstam et al. 2013a). The vision is thus sustainable landscapes. To apply the “landscape laboratory” design (e.g., Kohler 2002) the “Forest-Landscape-Society” research group at the Swedish University of Agricultural Sciences’ School for Forest Management received grants during the periods 2005–2007 and 2008–2012 from the Marcus and Amalia Wallenberg Foundation for the project “Connecting forests, people and markets in Europe’s East and West: adaptive governance and assessment of economy, environment, and society at multiple scales and levels.” Specifically, the aim of the project was to encourage the triple skills of (1) understanding the needs of landscapes’ stakeholders in different cultural contexts, (2) producing relevant knowledge, and (3) communicating it at different levels of society.

Realizing these ambitions requires time. We began in 2005 with the aim to develop our group’s ability to apply transdisciplinary research. This collaborative approach can be defined as problem solving that integrates researchers from multiple disciplines, and practitioners from different sectors at multiple governance levels. To understand bridges and barriers to implementing the vision of sustainable landscapes in Europe’s East and West, the project was based on collaboration with non-academic actors and stakeholders at multiple levels, including communicators in media, and with organizations involved in applied research, development, and education.

This special issue of AMBIO with 11 original articles illustrates this research journey (Table 1). We first present a systematic framework in seven steps based on multiple landscapes as case studies, collaboration among academic and non-academic actors and stakeholders, as well as communication with and dissemination to society at large (I). Next, to make effective use of multiple landscapes, or social–ecological systems, as replicated case studies by careful sampling in gradients that represent variation in biophysical, anthropogenic, and intangible dimensions of landscapes, we highlight the opportunity of bridging the different schools of landscape research in geography (II). Using the Bergslagen region in south-central Sweden as an example, we show the role of landscape history for the sustainability state of contemporary ecological and social systems (III). As an example of the fourth step, mapping stakeholders, use and non-use values, products and land use, we use a large forest management unit in Sweden and one in the Russian Federation to analyze how sustained yield forestry is defined and implemented (IV). To illustrate the importance of institutions, policies, and governance systems, we assess to what extent UNESCO’s Biosphere Reserve concept and its core functions are captured in national legislations in Ukraine and Sweden, and show how different political cultures have developed different implementation approaches (V). In a block of three articles, we then illustrate the opportunities and challenges of deriving indicators and measurable variables for ecological (VI), economical (VII), as well as social and cultural (VIII) sustainability dimensions. In a block of two articles, we illustrate the need for assessment or evaluation of sustainability and sustainable development processes by combining knowledge about policies, rules, and norms on the one hand, and the state and trends in ecological systems (IX), and adaptive governance, knowledge production, and learning in social systems (X) on the other. Finally, together with a group of researchers in different phases of learning the practice of transdisciplinary research, we analyze the barriers and bridges to integrative knowledge production and collaborative learning for problem solving (XI).

**Table 1:** Structure of the 11 articles in AMBIO 2013:2

	Description	Article	References
Overview	Seven-step approach	I	Angelstam et al. (2013a)
Step 1	Identify a suite of landscapes as case studies	II	Angelstam et al. (2013b)
Step 2	Study the landscape history	III	Angelstam et al. (2013c)
Step 3	Map stakeholders, use and non-use values, products, and land use	IV	Elbakidze et al. (2013a)
Step 4	Analyze institutions, policies, and governance system	V	Elbakidze et al. (2013b)
Step 5	Measure ecological, economic, social, and cultural sustainability	VI VII VIII	Richnau et al. (2013) Elbakidze et al. (2013c) Axelsson et al. (2013a)
Step 6	Assess sustainability dimensions and governance	IX X	Angelstam et al. (2013d) Axelsson et al. (2013b)
Step 7	Comparisons and syntheses	XI	Angelstam et al. (2013e)

Apart from publishing the research articles in peer-review journals, including this special issue of AMBIO, we have established productive collaboration with stakeholders from private, public, and civil sectors at multiple levels in Europe’s East and West. Our collaboration with stakeholders has resulted in the creation of the NGO Sustainable Bergslagen in Sweden—an emerging partnership working toward sustainable regional development (Axelsson et al. 2013b). Funding from different organizations (the Swedish Ministry of Environment, the European Union, the Swedish International Development Agency and others) supports our efforts to develop networking and neighborhood collaboration among researchers and practitioners toward sustainable landscapes in the Barents and Baltic Sea Regions. To disseminate the results from our research, we have developed education material about sustainable forest landscape management in Europe’s East and West for students at forestry universities. Finally, to secure communication with and dissemination to policy-makers, practitioners, and the general public, we have developed a network of researchers, journalists, and communicators in Europe’s East and West, and a common communication platform (the web site http://www.euroscapes.org will be officially launched in spring 2013). The research team has also developed contacts with media in Sweden, Russia, and Ukraine.

## Stakeholder Perspectives at Multiple Levels

### Lennart Myhrman: Local and Regional Rural Development

In Bergslagen where I was born, live, and work, people have for centuries been dependent on the use of the surrounding forest landscape and its natural resources. As long as there was no competition between different ways to use this natural capital there were no conflicts. But when Finns, encouraged by the Swedish government, colonized the sparsely populated wilderness and began to use upland forests for slash and burn cultivation, local hunters and fishermen felt displaced. Peasants from the surrounding plains also claimed the right to use trees for house building and other types of products needed for everyday life. Conflicts among different users of the forest landscape have since always been present, even if the stakeholders and the issues change.

Handling of conflicts among different parties involves the development of mutual understanding of perspectives and needs. There is a need to combine each party’s knowledge in a collaborative learning process and to develop experience-based learning among local, regional, national, and global level stakeholders and initiatives aiming for sustainable development toward sustainability. This would support the creation of a more holistic approach to handle natural resource management. A prerequisite for this process is to map and learn about local stakeholder’s interests and values. Such mapping improves understanding of possible goals of sustainable development, thus it could be distinguished according to the different parties, economic, ecological, and social premises. I see this special issue of AMBIO as an important step toward a greater understanding of the processes of sustainable development and sustainable natural resource use.

### Alexandr Mariev: Russian Federal Forestry Agency

Russia’s forests make up nearly a quarter of Earth’s forest cover. Characterized by high natural and historic diversity, these forests form a strategic renewable resource. Today, in the Russian Federation the societal demand regarding the quality of forest management has increased. Numerous scientific conferences, public discussions in media, public hearings have highlighted that there is an urgent need to develop national forest policy based on collaboration among multiple stakeholders. The main directions of the national forest policy are (1) preservation of forests and increase of forest cover, (2) development of sustainable forest management, (3) creation of an effective forest economy, and (4) a good environment for human life and well-being.

Sustainable forest management is essential for economic and social development. The main request from the forest industry is to adapt forest legislation, including rights of forest users, to historic, economic and social conditions of forest landscapes and regions. To improve the economic performance of forestry, the forest cover should be maintained in landscapes. Special attention is required in the sparsely forested regions, where the creation of protective forests is often needed.

The Russian Federal Forestry Agency is also committed to the development of forestry in combination with agriculture. Such an integrated approach will create conditions for the revival of rural and forest villages, provide jobs and give opportunities for rural development. We are also working on creating opportunities for active public involvement in forest management, especially by local populations. Thus, there is a need to support public hearing and include public expertise of forest management, especially related to forest use and lease.

It is important to learn about experiences of implementing sustainable forest management in countries and regions with different natural, historical, and societal contexts. This issue of AMBIO clearly shows the need to consider both forest ecosystems and society, and their integration in landscapes.

### Tatiana Tyopenko: Barents Region Collaboration

Natural resources and new transport routes have strengthened the Barents Region’s strategic significance to Europe. The Barents Region is also known for its clean nature, extensive forest areas, rich natural resources, and the unique culture of its indigenous peoples. All these make the region attractive for investors. Promotion of responsible and sustainable economic activity, taking into account environmental issues, is vital to the region’s prosperity. Nobody can remain indifferent to the beauty of Europe’s last large intact forest landscapes located in northwestern Russia. Simultaneously, unique natural landscapes have for centuries been a key part of the lifestyle of the indigenous people of the Barents Region. Sustainable development requires a well-informed and balanced approach to planned action. This is only possible with the participation of all stakeholders, including government, business, and civil society. However, such a process is very complex. There is neither always a common understanding of problems, nor among different experts. Today, many municipalities lack well-educated professionals, and are thus not able to make appropriate and timely decisions needed for sustainable development.

There is thus need for new know-how, including experiences from other countries. I see a huge potential for the development of neighborhood collaboration based on the experiences of transdisciplinary research presented in this issue of AMBIO. This includes collaborative learning and collaboration, which has been initiated between municipalities in Sweden and the Republic of Komi with the aim to learn from each other, to develop comprehensive plans and to steer the development in their respective territories toward sustainability.

### Niels Elers Koch: International Union of Forest Research Organizations (IUFRO)

Started in 1892 as a small network of forest research institutions, IUFRO is today a global network for forest research and higher learning, representing about 15 000 scientists in more than 120 countries. Over the years, IUFRO has transformed from a network of natural scientists to one addressing the whole range of ecological, economic, and social aspects of forests and trees. The representation of social sciences in the network has significantly expanded and today—among others—includes aspects such as forest policy and governance, traditional knowledge, human health, and recreation.

Knowledge production and learning for sustainable landscapes involving forest and tree resources is central to IUFRO’s work. To address current policy aims related to sustainable landscapes in a holistic and multi-disciplinary way, IUFRO arranges about 70 annual meetings around the world. Many of these are organized jointly by several scientific disciplines. IUFRO is a member of the Global Partnership of Forest Landscape Restoration, uniting more than 100 organizations toward the promotion of restoring important landscapes for the benefit of society.

Learning for sustainable landscapes also requires adequate dissemination of scientific knowledge to stakeholders and society at large. Over the past decade IUFRO has therefore made science communication and science–policy interactions a priority. To this end, IUFRO informs global policy processes, promotes science–policy interactions through cooperation with regional policy platforms, and supports capacity building of scientists on best practices in science communication and science–policy interactions. The excellent research presented in this issue of AMBIO is in line with IUFRO’s dual approach on interdisciplinary research and science–society interaction. Beyond doubt, more on this is needed for the benefit of both forest landscapes and people.
